# The Pathology of Fever

**Published:** 1899-10

**Authors:** J. Clarence Johnson

**Affiliations:** Atlanta, Ga.; Professor of Physiology and Pathological Anatomy Atlanta College of Physicians and Surgeons


ATLANTA

### Journal-Record of Medicine.

Successor to Atlanta Medical and Surgical Journal, Established 1855, and Southern Medical Record, Established 1870.

VOL. I. OCTOBER, 1899. NO. 7.

BERNARD WOLFF, M.D., EDITOR.

M. B. HUTCHINS, M.D., BUSINESS MANAGER.

PUBLISHED MONTHLY. OFFICE 305 AND 306 FITTEN BUILDING.

### *ORIGINAL COMMUNICATIONS.*

### THE PATHOLOGY OF FEVER.

BY J. CLARENCE JOHNSON, M.D.,

Professor of Physiology and Pathological Anatomy Atlanta College of Physicians and Surgeons.

(*Continued from September Number.*)

If we withhold all food for a given number of hours the potential of the excreta and the heat which they eliminate as free caloric should be equal to the total loss of energy from the body, and should be manifested by a diminished activity of the general functions proportionate to the mechanical equivalent of the loss. Such is the case, notwithstanding the fact the temperature remains the same, and the balance of the vital economy is not disturbed. So, also, where an excess of food is taken, its own potential or heat units are employed to compensate the energy expended in the process of digestion and absorption and the other organic functions which place it in its proper relation to the body, and that not so used is made a part of the *vis viva* of the body.

In fever, because of the fact that less food is taken than is needed for repair, and of the growing inefficiency of the cells of the body to perform their vital functions, and because they themselves reduce in size, the total amount of heat in the body is markedly more than in a body of the same weight in health, and yet the diseased body has less kinetic energy. Thus, while the tissue metamorphosis of fever may be more active than in health, it is destructive rather than constructive, and the evolution of heat may be in part chemico-vital, yet the cells themselves are neither capable of generating or applying their pro rata of the food-product or appropriating their share of heat as vital force.

Admitting that a vitiated blood supply perverts the function and nutrition of a cell, and that the toxalbumin of the blood is incapable, alone, of producing the elevation of temperature, we have sufficient proof that fever is not the result of exaggerated physiological processes. But, granting all this, the question remains: if it is chiefly a chemical process, or a physical phenomenon, how is physiological influence so suspended, restrained or modified as to permit or contribute to the increased temperature?

As no physiological process is possible or perfect without the consistent operation of the three factors named—blood, nerves and cells—the nervous system must play an important rôle in the pathology of fever.

It has already been stated that irritation of an end filament of a nerve will produce an elevation of the temperature of the part it supplies by exciting dilatation of the vessels.

But if the causative relation of the nervous system, or the part which it plays in the production of fever, is confined to its influence over vascular action, further consideration of this factor would be unnecessary, because the same influence is exerted alike in secretion and excretion, which diminishes or increases according to the amount and character of the blood.

That the nervous system is actively engaged in pathogenesis of fever, there is no doubt; but its action is not limited to the vasomotor nerves, as the normal heat-production involves both the cerebro-spinal and sympathetic systems, not only in their specific functions, but as tissues of the body.

If it were true that the cause of fever operated solely through the vasomotor nerves by calling into play their contracting influence upon the arterioles, ergot would induce an elevation of the temperature. If it operated by inhibiting them or by stimulating the vaso-dilators, whiskey would also elevate the temperature, which it does not, but on the contrary usually lowers it. Morphine is an excitant in small doses, and it does produce, sometimes, a slight elevation of the temperature, but it at the same time checks secretion and retards osmotic action.

Strychnine slighly elevates the temperature, but it does so by increasing reflex excitability, and, in consequence, the general nutritive action of the body.

There are medicines which reduce the temperature by lessening cardiac action or arterial tension, as aconite and veratrum, but the lowering of the tension is due not to a reduction of nerve action, but to a conversion of its energy into excretory and secretory force. Besides, arterial tension depends upon something more than nerve action. It is influenced—we may say governed—by the general state and physiological functions of the body.

The heart beats 72 times per minute, and expends, at each left ventricular systole, 1.3 lbs. of energy. This energy is conserved by the elastic and muscular coats of the vessels, and is supplemented by the *vis a fronte* of cellular attraction. It is modified by the character and the amount of blood conveyed, as is witnessed in asphyxia, where the capillaries refuse to pass it on account of the excess of carbon dioxide which it contains.

Injection of cocaine produces a rise of temperature, and affects chiefly the vaso-motor system, but the "cocaine fever" is caused by a secondary increase of the general functions, for a toxic dose produces a fall of temperature, with inhibition of general functions.

One of the most interesting features of the relation, which the nervous system bears to the pathology of fever, is that exhibited in ague. In this there is a chill of no definite duration and indefinite occurrence. The cutaneous capillaries are contracted by the vasomotor nerves and the blood is driven to the internal organs, when the temperature is markedly elevated. Soon there is a relaxation of the peripheral vessels and the skin becomes hot and dry. The temperature rises higher; within a few hours, it declines to normal. After a lapse of 24 or 48 hours the attack recurs.

During the interval, there has evidently been no destruction or elimination of the plasmodium malariæ. But why does the fever disappear so rapidly and return so soon?

The administration of morphine just before an expected chill will often abort or prevent it. How it does it is not known, but that it directly or indirectly combats the operation of the cause is manifest. Applying the physiological action of morphine upon the nervous system, it is safe to infer that it obtunds the reflex sensibility and thereby prevents the discharge of contractile force through the vaso-motor nerves. Thus are the germs or their toxalbumins rendered unable to produce peripheral anemia and lessen the elimination of heat.

But this does not explain how the plasmodium malariæ or its toxines so impress the nervous system or the general functions as to produce the disturbance in the balance of heat generation and elimination which primarily leads to the occurrence of the chill and the rise of temperature.

The ultimate effect of malaria is upon the blood-producing organs of the body. The spleen becomes enlarged, and the liver, which is a fertile source of heat, as we have seen, suffers material change in texture and in function. The character of the blood itself is impaired. The red corpuscles reduce in size and number. With a view of determining the exact relation which the nervous system sustains to heat-production various experiments have been made, some of the results of which have been already cited.

Puncture of the corpus striatum in a rabbit is followed by a rise of rectal temperature which may last for several days. Lesions of the cerebral cortex and of the medulla oblongata in man is followed by the same result. So with electrical stimulation in these regions. Hence have arisen theories that there are "heat-controlling" centers in the brain. But in each of the experiments just named there is a decline of temperature as the irritation subsides.

It would appear, therefore, that these nerve cells, irritation of which produce a rise of temperature, are intimately related with the nerves that have most to do with the cellular action of the body. The consensus of opinion is in favor of this view. It is easy, then, to understand how any agent, which constantly irritates or excites the corpus striatum or medulla oblongata, as blood laden with noxious matter, would cause increased heat generation and retention by contracting the cutaneous capillaries, since the vaso-motor center is situated in the fourth ventricle. Yet if the irritation involves also the vaso-dilator nerves, or if section is made severing the vaso-motor from the periphery, puncture of the corpus striatum is not followed by fever, because the cutaneous capillaries are dilated and the balance of body heat is maintained.

These facts demonstrate what has already been said of the relation of the physiological state of the body to its temperature.

It is evident that the influence of the nervous system in the production of fever is governed by its relation to the general trophic and functional action of the body, and that its perversion must partake of or follow changes in these functions.

Having shown that perversion, neither of the blood, the nervous system, nor the cells, alone, is capable of producing abnormal temperature, let us seek to ascertain how perversion of them all can produce it. In this effort, we must not lose sight of the relation and mutual dependence of these factors in all physiological action, or forget that the blood and cells are so altered in their histological and chemical characters by the unformed ferments of bacteria, that they are, in a great measure, unfitted for the purposes of nutrition and function.

Reverting to the proposition, that whatever causes fever does so either by increased heat generation, deficient elimination or conversion into energy, we are brought to consider how these compensatory functions are maintained.

We have already stated that a body weighing 160 pounds, or 73 kilos, normally generates 7,286.4 units, or 3,312,000 small calories, within twenty-four hours. Within one hour, it generates 140 calories—140,000 small calories—or enough to raise the temperature of the body 5° F., or about 2.75 Centigrade. The caloric capacity of the body as compared to water, at 100, is about 90. Taking one heat unit or one-half calorie as the amount required to raise one pound of water 1° C., it would require 160 units, or 80 calories, in round numbers, to raise the temperature of the body 2° F., or 1° Centigrade. It would require 98½ times 80, or 7,880 units, or 3,940 calories, to heat the body sufficiently to give it a temperature of 98.5° F., or 37.5° C. Hence, the caloric capacity of the human body, weighing 160 pounds, at normal temperature, is equivalent to 7,880 thermal units, or 3,940 large calories. If enough heat is generated within one hour to raise the body temperature 5° F., then the combustion of oxidizable material must produce of free caloric 140, or 308 thermal units, else there must be another source of heat in the body.

Of the 3,312 large calories generated daily, only about one-fifth is used as kinetic energy. Part is employed in preserving the body temperature and the balance is set free. The channels of elimination are the skin, lungs and excreta.

Following is the amount and method of elimination by each:

| | | | |
|---|---|---|---|
| SKIN | By water | 400 | calories. |
| | By radiation | 750 | " |
| | By convection and conduction | 900 | " |
| | Total | 2,050 | " |
| LUNGS | By vapor | 400 | calories. |
| | Heating expired air | 70 | " |
| | Total | 470 | " |
| | By the excreta, viz.: — | | |
| | Urine and feces | 70 | calories. |

—*Stewart's Manual.*

The one-fifth used as energy is divided among the various organs for the performance of their nutritive and functional offices. The heart consumes, within 24 hours, 63,000 small calories, for the accomplishment of its systolic and diastolic action.

The respiratory muscles consume about 30,500 small calories. The skeletal muscles generate three or four times as much heat as they consume or expend as force in their contractions. The glands likewise contribute their share of heat and use their pro rata as kinetic energy.

Taking the heart as an example of an organ demonstrating the relation of heat and force, we will perceive that, all things else being equal, the temperature of the body depends, not only upon the amount of heat which it generates and sets free, but upon that also which is hidden or engaged as force. Thus the heart expends 27,000 kilogrammeters, which is the equivalent of 63,000 small calories. These heat units, or calories, are borrowed from the aggregate of the body heat, or potential—which is really possessed by the cells—and is converted into and employed as contractile force.

No force in the body can be lost, nor can the aggregate be reduced, save by overcoming the resistance of atmospheric pressure and the accomplishment of voluntary work, or by conversion into heat, in which form it may be set free. So the 1.3 pounds energy which the left ventricle expends in each systolic action, when its work in propelling the blood through the vessels is done—as does also that employed by the muscular and elastic coats—appears as heat, which supplements the potential of proximate principles, for the delivery of which the cells made the loan.

What has been said of the heart applies to every organ of the body. If the cells are thus furnished with more potential than they need or can utilize, by concerted physiological action, the excess is set free. If the physiological action of the cells is impaired, this and other functions are imperfectly performed, and they are unable to appropriate and conceal, as kinetic or potential energy, or to discharge, their accustomed share of heat.

We have seen that, in essential fever, the condition of the blood and tissues is directly opposed to normal physiological action. We have seen that combustion may be less than occurs in health. So, in the reaction of the febrile state, energy is changed to heat, and, with this conversion, there is a gradual loss of vital power. The heart beats faster and with less force, seeking to maintain a compensatory function, but its accomplishment falls short of its requirements, and the general organs suffer from the disturbed relation which this perverted action imposes. If this condition is not relieved, the heart soon pays the penalty for its helpless and blameless inefficiency, and, unsupported by the wonted contribution from the cells, ceases its operations, and death claims the victory. But, if compensatory functions are maintained, if the balance of the vital economy is preserved, or its equilibrium is not too far disturbed, if the *vis medicatrix naturæ* is upheld and aided by art, properly applied, the battle turns in favor of life and health, the foe is driven from its stronghold, the smoke and ashes of the conflict are cleared away, and the various organs resume their normal action and relation.

By recapitulation, we will observe the following facts most pertinent to the pathology of fever:

*First:* The human body is a complex whole, composed of independent physiological units, each of which embodies the general and specific properties of the elementary matter of which it is formed, and by virtue of which it has function and relation.

*Second:* In the maintenance of this relation and accomplishment of this function, there is a constant change of state which defines the metabolism of the cells, and from which evolves the heat that determines the body form and temperature, by its latent, specific and potential capacity.

*Third:* The balance of the vital economy is preserved by the introduction, digestion and appropriation of proximate principles as food, with caloric sufficient to supply the force expended in metabolic action, and by the elimination of products rendered effete by this process.

*Fourth:* The factors engaged in the performance of all physiological action, and, hence governing the production and disposition of heat, viz.: the cells, blood and nervous system, are mutually dependent. So, any cause of abnormal temperature, or fever, must operate through one or all of these factors, and, primarily, or secondarily, each must participate in the morbid process.

*Fifth:* The character of the blood, the condition of the cells, and the action of the nervous system are not only insufficient to explain the phenomena of fever as a physiological product, but are opposed to its production after the usual manner of thermogenesis.

*Sixth:* The sum of evidence favors and supports the conclusion that the relation of the chemical and physiological forces, in their action and reaction as physiological agents, is disturbed by the general conditions directly induced by the exciting cause, in consequence of which osmotic interchange is inhibited, anabolism is diminished, physiological becomes subservient to chemical action, and physical energy, losing thus its exercise, is reconverted into heat, which retards, to the extent of its mechanical equivalent, the secretory, excretory, and other functions of the body. The amount of body heat increases, the temperature rises and remains above the normal.

Fever, then, is not an entity, not a disease, but a part and product of a morbid process—the physiology of inconsistent thermogenic and thermotaxic action, the symbol of perverted function, as delirium is of disordered intellection, or convulsions of incoordinate muscular contraction.

